# Advances in phage therapy for *Acinetobacter baumannii*: mechanisms and applications

**DOI:** 10.3389/fmicb.2026.1839739

**Published:** 2026-06-25

**Authors:** Sijia Cheng, Siwei Zhou, Xiaoyi Wang, Zhaoxi Yu, Lei Liu, Jie Liu, Lihua Qi

**Affiliations:** 1 Clinical Laboratory Department, Seventh Medical Center, PLA General Hospital, Beijing, China; 2 Division of Life Sciences, Faculty of Science, The Chinese University of Hong Kong, Hong Kong, Hong Kong SAR, China; 3 Comprehensive Treatment Department, Second Medical Center, PLA General Hospital, Beijing, China

**Keywords:** *Acinetobacter baumannii*, genetic engineering, mechanism, phage, therapy

## Abstract

*Acinetobacter baumannii* poses a severe global health threat due to its extensive multi-drug resistance. This review explores the evolving role of phage therapy as a promising alternative against multi-drug resistant *Acinetobacter baumannii* infections. We reviewed the latest key mechanisms by which phages exert their therapeutic effects, including direct lysis, biofilm disruption via depolymerases, resensitization of resistant strains to antibiotics through receptor-mediated fitness trade-offs, and the action of phage-derived enzymes such as endolysins. Recent preclinical studies have demonstrated robust efficacy, while clinical case reports and ongoing trials highlight both the potential and challenges of compassionate phage use, including emergence of phage resistance and variable patient responses. Advances in pharmacokinetic optimization, including PEGylation to enhance circulation and immune evasion, are discussed alongside synergistic phage-antibiotic combinations and novel delivery systems such as hydrogel formulations for topical applications. The review further examines emerging strategies in phage engineering and synthetic biology aimed at overcoming host-range limitations and resistance development, including chimeric lysins with enhanced outer membrane penetration and photosensitizer-conjugated phages for biofilm eradication. Finally, we highlight emerging strategies in phage engineering and synthetic biology aimed at overcoming host-range limitations and resistance development, so as the role of artificial intelligence in cocktail design and personalized therapeutics.

## 1 Introduction

Among the latest key pathogens cataloged in the World Health Organization (WHO) bacterial priority pathogens list, 2024, carbapenem-resistant *Acinetobacter baumannii* (CRAB) is classified in the critical priority group, underscoring the serious and escalating threats to global health (World Health Organization, 2024). Known for its extensive antibiotic resistance, this Gram-negative bacterium has emerged as a leading cause of devastating healthcare-associated infections, including ventilator-associated pneumonia, bloodstream infections and wound infections, particularly in Intensive Care Units (ICU) worldwide (Jiang et al., 2022). Moreover, the emergence of multi-drug-resistant (MDR) *Acinetobacter baumannii* (*A. baumannii*) underscores the critical limitations of conventional antibiotics. As emphasized by a recent genomic surveillance study, a dominant epidemic clade (Clade 2.5.6) has undergone a stepwise accumulation of resistance genes, rendering it nearly universally resistant to most front-line therapies (Li S. et al., 2025). This escalating crisis necessitates the urgent exploration of alternative strategies. Phages are viruses that specifically infect and lyse bacterial cells. They offer a highly targeted antimicrobial strategy capable of combating MDR infections without disrupting the host microbiota. Although phages have been considered as therapeutic agents since the early 1920s (Tu et al., 2023), their therapeutic application has been overshadowed by the emergence of broad-spectrum antibiotics. Nowadays, phage therapy has emerged as a promising solution to combat the growing crisis of MDR *A. baumannii* infection (Kim et al., 2025). There have been several international centers actively engaged in implementation of phage therapy, and recent case series have reported encouraging success rates in patients receiving personalized, compassionate phage therapy for difficult-to-treat infections (McCallin et al., 2019). In this review, we provide an overview of the current application status of phage therapy in *A. baumannii*, and by delving into the latest research findings on the role of phages, we aim to provide perspectives and development directions for phage therapy against *A. baumannii* in the near future.

## 2 Literature search strategy

Relevant literature was identified through targeted searches in PubMed and Web of Science, using combinations of keywords including “*Acinetobacter baumannii*”, “phage therapy”, “endolysin”, “depolymerase”, “phage-antibiotic synergy”, and “biofilm”, among others. Priority is given to research articles, clinical case reports, preclinical studies, and reviews published within the last 5 years, while also including earlier milestone studies. The final reference list was curated based on relevance to the mechanistic and clincial transition themes of this review.

## 3 Preclinical and clinical studies

### 3.1 *In vivo* and animal model studies

Animal models have been instrumental in validating the efficacy of phage therapy against *A. baumannii* infections. Invertebrate models, particularly *Galleria mellonella*, serve as rapid screening platforms. For example, phage Ab_WF01 significantly increased the survival of larvae infected with carbapenem-resistant *A. baumannii* (CRAB) within 48 h (Wang Z. et al., 2024). Similarly, the activity of phage vB_AbaP_AGC01 and its synergy with antibiotics were confirmed in this model (Grygorcewicz et al., 2020). A three-phage cocktail improved *G. mellonella* larval survival against extensively drug-resistant *A. baumannii* to 85% at day 1 and 60% at day 7 post-infection (*p* < 0.0001) (Sawaengwong et al., 2026).

Murine models provide more physiologically relevant insights into therapeutic and prophylactic potential of phages against *A. baumannii*. Phage Ab_WF01 rescued 60% of CRAB-infected mice over 7 days (Wang Z. et al., 2024). In pulmonary infection models, a single intratracheal dose of phage vB_AbaM_Acibel004 reduced bacterial load and inflammation without adverse effects (Wienhold et al., 2021). Prophylactic administration of phage vB_AbaM_P1 in a neutropenic rat model significantly lowered mortality and lung bacterial burden (Li et al., 2024a). Moreover, a two-phage cocktail (vB_AbaM_P1 and vB_AbaM_DP45) effectively reduced lung bacterial load, improved survival, and modulated immune responses in a rat pneumonia model (Li et al., 2024b). A novel virulent phage, vB_AbaS_qsb1, administered intratracheally at 3 × 10^9^ PFU/mouse in a murine pneumonia model, provided at least 50% protection against *A. baumannii*-induced infection, significantly reducing bacterial loads (from 10^8^-10^10^ CFU/g to 10^6^ CFU/g by 48 h) and TNF-α levels in lung tissue, while maintaining high phage titers (10^10^ PFU/g) for 7 days (Wang et al., 2025a). In a rat burn wound model, the lytic phage vB-AbauM-Arak1 markedly reduced lesion area and accelerated healing over a 14-day observation period (Ghaznavi-Rad et al., 2022). Additionally, a zebrafish embryo bath immersion model demonstrated that phage treatment targeting the most virulent *A. baumannii* capsule types (K2, K9, K32, K45) increased average survival from 35.2% to up to 74.1%, supporting the utility of this model for rapid *in vivo* efficacy assessment (Neto et al., 2023).

These studies demonstrate the robust and reproducible efficacy of phage therapy across a spectrum of preclinical infection models, highlighting its potential for treating CRAB infections.

### 3.2 Case reports, institutional support, and environmental applications

Several recent case reports have documented the compassionate use of phage therapy against CRAB infections, primarily in critically ill patients with limited antibiotic options. Table 1 summarizes key clinical cases, patient characteristics, pathogen spectrum, treatment regimens, and outcomes.

**Table 1:** Key information from recent studies on phage therapy for *A. baumannii* infections.

Case (first author, year)	Patient profile	Infection type	Pathogen spectrum	Phage regimen	Key clinical outcome
(Schooley et al. 2017)	68 M, diabetes, necrotizing pancreatitis	Disseminated (abscess, bloods)	Monomicrobial (MDRAB)	IV phage cocktails (ΦPC, ΦIV, then ΦIVB) + antibiotics	Improved (resistance emerged, resensitized to minocycline)
(Tan et al. 2021)	88 M, COPD, pneumonia	Pulmonary	Monomicrobial (CRAB)	Nebulized Ab_SZ3 + tigecycline/polymyxin E	Improved
(Wu et al. 2021)	62–81 M, 4 critical COVID-19 patients	Pulmonary	Polymicrobial (CRAB + *Candida spp*., CRKP, etc.)	Pre-optimized 2-phage nebulized cocktail (ΦAb124+ΦAb121)	2 improved, 2 died
(Eales and Tam 2022)	VAP	Pulmonary	Monomicrobial (CRAB)	Nebulized phage + cefiderocol	Improved
(Qu et al. 2024)	71 F, type 2 diabetes, ependymoma, pneumonia	Pulmonary	First round monomicrobial (XDRAB), second round polymicrobial (XDRAB + *P. aeruginosa*)	Inhaled phage BA3 + antibiotics (two rounds)	First round effective; second round with phage resistance
(Lin et al. 2025)	80s F, hypertension, COPD, bronchial asthma	Pulmonary	Monomicrobial (XDRAB)	Nebulized phage + polymyxin B, amikacin, fosfomycin	Improved

CRAB, carbapenem-resistant *A. baumannii*; XDRAB, extensively drug-resistant *A. baumannii*; MDRAB, multidrug-resistant *A. baumannii*; VAP, ventilator-associated pneumonia; COPD, chronic obstructive pulmonary disease; COVID-19, coronavirus disease 2019; CRKP, carbapenem-resistant *Klebsiella pneumoniae*.

Such reports highlight the ability of phage therapy to clear resistant pathogens, while also underscoring issues such as resistance emergence and heterogeneous patient responses (Muñoz-Egea et al., 2026). It is important to acknowledge that the current clinical evidence base for phage therapy specifically against CRAB pneumonia or bloodstream infections remains insufficient. The available literature primarily consists of single case reports and small, uncontrolled cohort studies employing heterogeneous protocols. To date, few phase III randomized controlled trials specifically evaluating phage therapy for CRAB infections have been completed. The highest available level of evidence remains limited to case reports and small case series (equivalent to Level IV-V evidence according to the Oxford Center for Evidence-Based Medicine (OCEBM) 2011 levels of evidence), which are subject to publication bias and lack of control groups. Therefore, definitive conclusions regarding efficacy and safety cannot be drawn. Well-designed randomized controlled trials are urgently needed.

As shown in Table 1, polymicrobial infections are common in these patients. CRAB frequently co-exists with other pathogens such as *Candida albicans*, carbapenem-resistant *Klebsiella pneumoniae*, or *Pseudomonas aeruginosa* (*P. aeruginosa*). These observations raise several important considerations: phages are highly species-specific and do not cross-kill unrelated pathogens; concurrent antibiotic adjustments may confound efficacy assessments; polymicrobial biofilms may exhibit altered susceptibility to phages; and the presence of multiple pathogens complicates interpretation of microbiological clearance data. Future case reports should systematically document co-infecting organisms, and preclinical models incorporating polymicrobial infections are urgently needed to guide therapeutic strategies that reflect authentic clinical complexity.

Institutional frameworks have been crucial for advancing phage therapy. Several specialized phage therapy centers have been established worldwide to facilitate clinical access and research. These include the Center for Innovative Phage Applications and Therapeutics (IPATH) at UC San Diego (USA), the Belgian Phage Bank (Belgium), the Ludwig Hirszfeld Institute's Phage Therapy Unit in Wrocław (Poland), the Eliava Institute (Georgia), the Shanghai Phage & Drug Resistance Institute (China), and the Shenzhen Phage Bank (China) (McCallin et al., 2019). In China, translational efforts are further supported by companies like Qingdao Nuobeite, TargetPhage BioMed, and PhageGene.

Beyond clinical use, phages have been explored for environmental decontamination and infection prevention. Certain phages remain active in the presence of chemical disinfectants, and their combined use enhances the removal of bacterial biofilms from hard surfaces (Chen et al., 2024). Integrating lytic phages into probiotic cleaning hygiene systems (PCHS) could improve pathogen reduction and control healthcare-associated infections (D'Accolti et al., 2023).

### 3.3 Ongoing clinical trials and regulatory challenges

Combating CRAB will require not only new antibiotics but also innovative strategies. According to a report in 2025, six non-traditional antimicrobial approaches are being evaluated in Phase 1 or 2 trials, including two monoclonal antibodies, a defined phage therapy product, an immune-modulator, a microbiome-modulator, and an engineered cationic antimicrobial peptide (Kontogiannis et al., 2025). Several clinical trials are evaluating the safety and efficacy of phage therapy for *A. baumannii* and other pathogens. In 2025, a randomized double-blind study on the treatment of diabetic foot ulcers (DFUs) using a topical phage cocktail (TP-102) targeting *Staphylococcus aureus, P. aeruginosa*, and/or *A. baumannii* has shown that TP-102 is more effective than placebo in reducing the microbial load of target pathogens and was associated with a higher wound closure rate, with excellent tolerability and no related adverse events (Nir-Paz et al., 2025).

Despite these promising clinical data, regulatory hurdles significantly limit the widespread adoption of phage therapy (Zalewska-Piatek, 2023). The regulatory landscape for phage therapy varies considerably across jurisdictions. In the United States, phages are regulated as biological products by the FDA under the Center for Biologics Evaluation and Research (CBER), with access primarily through standard Investigational New Drug (IND) applications, expanded access INDs for compassionate use, or single-patient INDs for emergency treatment (Niazi, 2025; Center for Biologics Evaluation Research, 2021). In the European Union, phages are classified as biological medicinal products under Directive 2001/83/EC, but individual member states have adopted divergent implementation approaches: Belgium has pioneered a magistral preparation framework that allows patient-specific phage compounding without full GMP certification, while other countries rely on experimental use exemptions or compassionate use programs (Marino et al., 2025; Pirnay et al., 2018). A recent international survey of regulators from 21 countries revealed that nearly half of the respondents had no prior involvement in phage-related regulatory processes in the past decade, and 32% were uncertain whether phages had ever been used therapeutically in their country. Moreover, 27% of regulators acknowledged that addressing single-use cases consumes a significant amount of their resources compared to other regulatory applications (Alvarez et al., 2025a). These findings underscore a critical gap in regulatory awareness and infrastructure that must be addressed to facilitate clinical access.

A fundamental tension in phage therapy regulation exists between the traditional pharmaceutical paradigm of standardized, industrially manufactured products and the personalized, adaptive nature of phage therapy (Marino et al., 2025). Industrial development requires full GMP compliance, extensive preclinical and clinical trials, and marketing authorization—a process that is time-consuming, costly, and ill-suited to the strain-specificity of phages. In contrast, the personalized magistral approach, exemplified by Belgium's framework, allows rapid adaptation to patient-specific bacterial isolates without market authorization and raises questions about quality consistency (Marino et al., 2025; Pirnay et al., 2018). Recognizing this challenge, experts have increasingly called for adaptive regulatory frameworks that differentiate between industrial phage products and personalized preparations (Marino et al., 2025). The European Pharmacopoeia has recently adopted a new general chapter on phage therapy medicinal products (Chapter 5.31), representing an important step toward harmonized quality standards across Europe (European Pharmacopoeia, 2025).

Beyond regulatory pathways, the emergence of phage-bacteria co-evolution leading to phage resistance complicates trial design and outcome assessment (Oromí-Bosch et al., 2023). Recent initiatives, such as expanded access programs, are beginning to define therapeutic pathways. A survey of clinicians with phage therapy experience identified regulatory guidance as the second most pressing concern (ranked as the top concern by 28% of respondents), following evidence for clinical use (Alvarez et al., 2025b). Both regulators and clinicians have expressed strong support for establishing international or national phage therapy registries, which were considered highly beneficial for tracking outcomes and adverse events (Alvarez et al., 2025a,b). Such registries could provide valuable evidence to complement controlled trials and inform regulatory decision-making.

International regulatory harmonization, validated phage libraries, and standardized manufacturing remain essential to transition phage therapy from compassionate use to routine clinical practice. Proposed reforms include risk-based GMP standards for expanded access, adaptive trial designs that allow sequential cocktail switching, and multi-agency advice frameworks to guide developers (Niazi, 2025; Marino et al., 2025; Alvarez et al., 2025a). Without these advances, the therapeutic potential of phage therapy against CRAB and other MDR infections will remain limited.

## 4 Mechanisms of phage therapy against *A. baumannii*

### 4.1 Capsular polysaccharide degradation and host specificity determination

**Figure 1:**
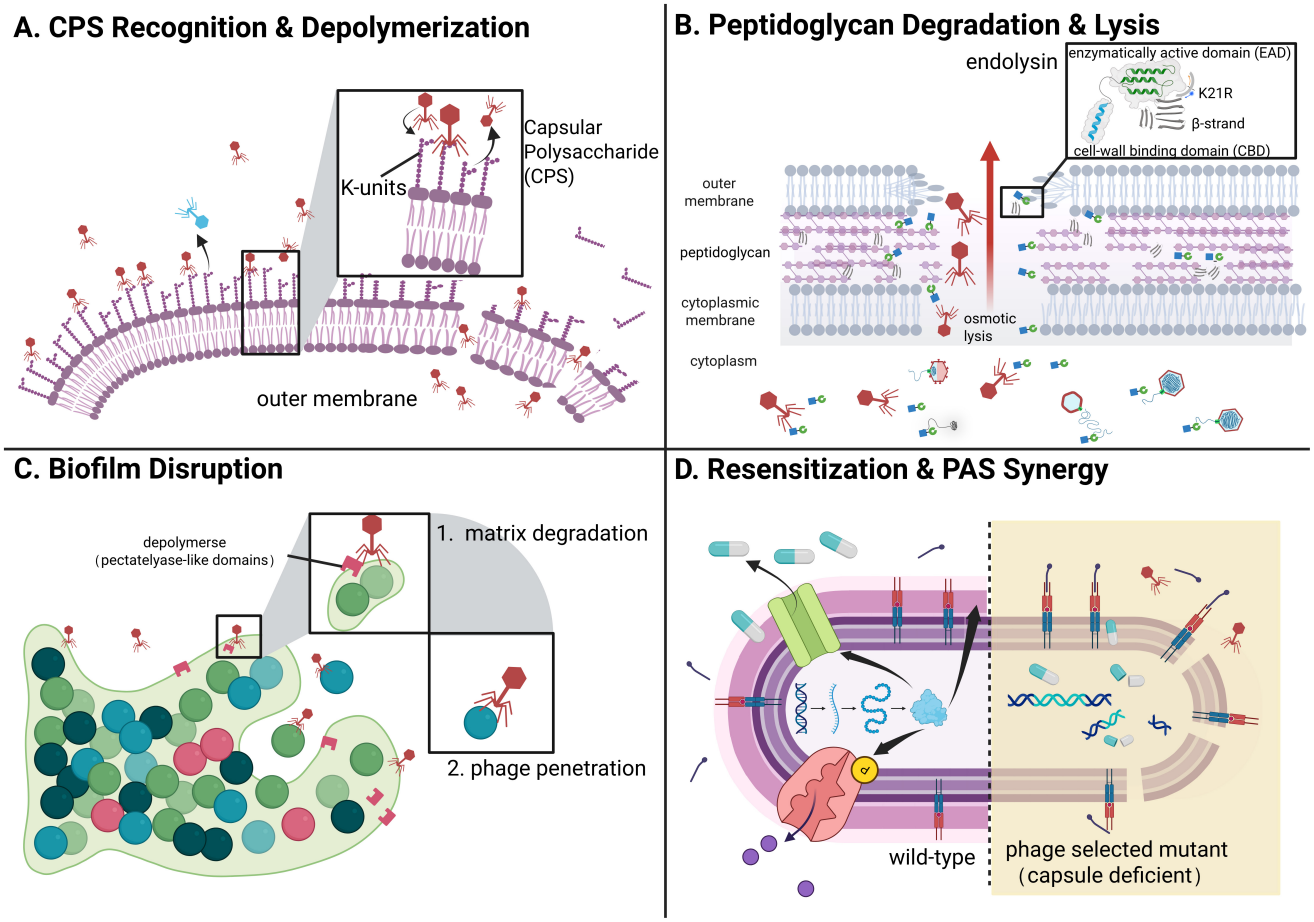
Mains mechanisms of phage therapy against *Acinetobacter baumannii*. **(A)** Capsular polysaccharide degradation and host specificity determination. **(B)** Bacteriolysis via phage-encoded endolysins. **(C)** Disruption of biofilms to overcome physical barriers. **(D)** Resensitization to antibiotics and complement system. Figure was created with BioRender.com.

The CPS is a major virulence factor for *A. baumannii*, forming a thick protective layer that shields the bacterium from environmental stress and antimicrobial agents, and simultaneously serving as the primary receptor for capsule-specific phages (Evseev et al., 2024; Islam et al., 2024). The extensive structural diversity of CPS, which can even incorporate novel sugar residues via prophage-derived genetic insertions, underscores the complexity and specificity of phage-host interactions (Zou et al., 2024) (Figure 1A). Phage adsorption is mediated by tail spike proteins with depolymerase activity, which specifically recognize and cleave CPS, thereby determining phage host range and facilitating bacterial lysis (Evseev et al., 2024; Timoshina et al., 2023a). This mechanism is hydrolytic, breaking the linkage between repeating K-units to release oligosaccharides or monomers, thereby stripping the bacterial cell of its capsule and facilitating phage adsorption and subsequent lysis (Drobiazko et al., 2022; Kasimova et al., 2024). Their high specificity arises from precise structural recognition, meaning a depolymerase typically targets only one or a few closely related CPS structures. Numerous studies have elucidated the structure-function relationship of these enzymes and their corresponding CPS targets. For example, phages targeting K9 CPS (e.g., AM24, BS46, APK09) can also cleave the structurally similar K70 CPS (Kasimova et al., 2023). Also, minor structural variations can generate or abolish susceptibility. Loss of a β-D-GlcpNAc side chain in K3-v1 CPS confers sensitivity to phage APK37.1, while native K3 CPS remains resistant (Timoshina et al., 2023a). The K239 CPS consists of heptasaccharide repeating units that are structurally related to those of the K86 CPS. However, the glycosidic linkages joining these units differ due to the presence of a distinct Wzy polymerase in the K239 biosynthesis gene cluster. This structural difference renders K239 resistant to the APK86 phage that effectively targets and cleaves K86 CPS (Kasimova et al., 2024).

Characterized depolymerases now target numerous K-types, including K1, K9, K14, K16, K37, K86, K102, K127, and K128 (Kasimova et al., 2024; Timoshina et al., 2023b). These enzymes can be sourced from lytic phages or prophages within bacterial genomes, serving as a natural reservoir for polysaccharide degradation (Drobiazko et al., 2022). Therapeutically, recombinant depolymerases alone can decapsulate bacteria, enhance serum killing, and reduce mortality in infection models (Timoshina et al., 2023b; Drobiazko et al., 2022). Depolymerase-mediated capsule degradation is central to phage infectivity and host specificity, offering a promising basis for developing targeted enzyme-based therapeutics or rational phage cocktails against MDR *A. baumannii*. Notably, recent evidence indicates that not all *A. baumannii* phages rely on CPS as a receptor. A newly described phage genus infects via a non-capsular target, with the capsule actually impeding rather than facilitating infection (Kenney et al., 2025). Such non-capsule-dependent phages may expand the therapeutic arsenal and complement capsule-targeting agents in cocktails to circumvent capsule-mediated resistance.

### 4.2 Bacteriolysis via phage-encoded endolysins

Phage endolysins are enzymes that degrade the bacterial peptidoglycan (PG) layer from within during the lytic cycle, leading to osmotic lysis and host cell death. When applied exogenously as purified proteins, they represent a highly specific antibacterial strategy against CRAB, offering a promising alternative to conventional antibiotics (Chu et al., 2022; Lai et al., 2013). Structurally, endolysins typically exhibit a modular architecture consisting of two functional domains: one or more enzymatically active domains (EADs) that cleave specific bonds within the PG, and a cell wall-binding domain (CBD) that confers target specificity (Euler et al., 2023) (Figure 1B). This modular design allows for both precise targeting and efficient PG degradation. Functional studies have identified several endolysins with potent activity against MDR *A. baumannii*. For instance, LysAB1245, a lysozyme-like endolysin derived from phage T1245, demonstrates broad-spectrum and rapid bactericidal activity against diverse capsular types. It also maintains stability across a wide range of temperatures (4–70 °C) and pH levels (4.5–10.5), underscoring its potential therapeutic utility (Soontarach et al., 2024). Similarly, genome mining of *A. baumannii* ATCC 17978 led to the discovery of LysAB3, an endolysin exhibiting significant PG-lytic activity against most *Acinetobacter spp*. with species-specificity, highlighting the feasibility of deriving therapeutic endolysins directly from bacterial genomes (Lai et al., 2013).

Despite these advantages, endolysins have several limitations. Their large size and hydrophilic nature limit outer membrane penetration, necessitating engineering or permeabilizers (Li Y. et al., 2025; Ullah et al., 2025). As foreign proteins, they may elicit neutralizing antibodies, reducing efficacy upon repeated dosing (Choi et al., 2026). Their *in vivo* half-life is typically short, requiring frequent administration. Additionally, bacteria can evolve resistance via peptidoglycan modifications, an underappreciated but emerging concern (Oromí-Bosch et al., 2023).

### 4.3 Disruption of biofilms to overcome physical barriers

Biofilm formation is a key virulence factor and a major therapeutic obstacle in *A. baumannii* infections. These structured communities, encased in an extracellular polymeric substance (EPS) matrix, confer extreme resistance to antibiotics and host immune defenses (Mendes et al., 2023). The clinical challenge is substantial, as over 66% of *A. baumannii* isolates are biofilm-producers, with a significant association observed between MDR and robust biofilm production (Gautam et al., 2023). Phages and endolysins both exhibit biofilm reduction effects. PlyF307 similarly reduced bacterial density by 1.6 log_10_CFU in an *in vitro* catheter biofilm model and by 2 log_10_CFU in a murine model (Lood et al., 2015). For endolysins, LysAB3 and LysAB2-KWK have demonstrated inhibition rates of 95.8% and approximately 40%, respectively, against *A. baumannii* biofilms (Zhang et al., 2018; Chen et al., 2021).

Phage therapy combats biofilms through multiple mechanisms, with enzymatic degradation of the EPS matrix being a primary strategy. Depolymerases of phages recognize and degrade key polysaccharide components of the biofilm matrix, such as capsular or slime polymers, thereby disrupting the biofilm's structural integrity and facilitating phage access to embedded bacterial cells (Grygiel et al., 2024) (Figure 1C). For example, phage vB_AbaM_AB4P2 possesses a tail fiber protein with a pectin lyase-like domain that directly contributes to biofilm-clearing efficacy, achieving a 33% reduction of pre-formed biofilms on PVC catheters within 2 hours (*p* < 0.05) (Su et al., 2025). Similarly, phage vB_AbaM-SHI not only inhibits *A. baumannii* biofilm formation, but also exhibits intrinsic bacteriolytic activity, particularly under high-density bacterial conditions like biofilm. Genomic and structural analyses indicate that its endolysin lacks typical holin/spanin genes and possesses a unique arginine substitution (K21R) that introduces additional β-strands, potentially enhancing autonomous cell wall degradation and contributing to its independent anti-biofilm and bactericidal effects (Jiang et al., 2024). The degradation of the biofilm matrix not only directly lyses embedded bacteria but also can re-sensitize them to conventional antibiotics by breaking down the physical barrier that impedes drug penetration (Mendes et al., 2023).

The efficacy of phage-mediated biofilm disruption is highly dependent on the maturation stage of the biofilm. Biofilm development in *A. baumannii* typically proceeds through five stages: initial reversible attachment, irreversible attachment, EPS production and growth, maturation, and finally dispersal. Early-stage biofilms, characterized by loosely attached bacterial cells and a nascent EPS matrix, are generally more susceptible to phage penetration and lysis. In contrast, mature biofilms feature a dense and structured EPS matrix along with metabolically dormant persister cells in deeper layers, which substantially hinder phage diffusion and replication (Mendes et al., 2023; Wong et al., 2021; Costerton et al., 1995; Buret et al., 2019). Quantitative data from phage-derived enzyme studies support this stage-dependent susceptibility: the endolysin Abp013 achieved a 2.65 log10 CFU reduction (99.78%) in 3-h biofilms, but only a 0.827 log10 CFU reduction (85.13%) in 24-h mature biofilms of *A. baumannii* (Chu et al., 2022). For intact phages, however, standardized quantitative data of reductions is still limited. Phage vB_AbaP_WU2001 achieved 48.72% inhibition of biofilm formation and 78.82% degradation of mature biofilm (Wintachai et al., 2022). Phage AB1801 showed 66% inhibition of biofilm formation and 70% degradation of pre-formed biofilm (Wintachai et al., 2019). These results illustrate that the effect of biofilm reduction varies considerably across studies due to differences in experimental conditions (e.g., biofilm age, multiplicity of infection, phage concentration, and measurement methods).

Worthy of note, despite abundant *in vitro* evidence, no *in vivo* study to date has documented the use of phages or their derived enzymes to disrupt pre-existing biofilms in animal models. Whether these agents can effectively penetrate and eradicate *A. baumannii* biofilms *in vivo* remains to be validated in future investigations.

### 4.4 Evolutionary trade-offs and active anti-phage defense systems

Phage infection can also induce genetic alterations that indirectly restore antibiotic susceptibility, a phenomenon known as evolutionary trade-off. This resensitization primarily occurs through phage-induced physiological or genetic alterations in bacteria, often resulting in potent phage-antibiotic synergy (PAS).

The most commonly observed mechanism involves mutations that alter or down-regulate surface structures serving as phage receptors, such as CPS or outer membrane proteins (Figure 1D). Phage-resistant *A. baumannii* mutants, selected via loss-of-function mutations in capsule biosynthesis genes, became avirulent and were re-sensitized to β-lactam antibiotics as well as human serum complement (Gordillo et al., 2021). Nayak et al. reported that phage-resistant strains exhibited significant down-regulation of *omp* genes, particularly *ompA*, which was associated with reduced phage adsorption, deviant growth rates, biofilm-forming capacities, and, crucially, re-sensitization to antibiotics the strains had previously resisted (Nayak et al., 2025). Resistance to capsule-targeting phages frequently leads to capsule-deficient mutants. These mutants demonstrate collateral sensitivity to colistin, as the loss of the capsule compromises envelope integrity and enhances antibiotic penetration (Wang et al., 2021). This “evolutionary trade-off” is further supported by Manley et al., who found that phage resistance in *A. baumannii* incurred a clear cost to virulence, suggesting a general fitness defect that can be exploited therapeutically (Manley et al., 2024). This fitness cost extends to non-capsule-receptor phages as well. *A. baumannii* populations acquiring tolerance to Canalvirus phages exhibited a marked decrease in biofitness ex vivo. When cultured in 50% human ascites, which is a host-relevant environment containing complement and other bactericidal factors, these phage-resistant pools showed significantly reduced survival compared to the wild-type parent strain, with five out of six pools yielding no recoverable CFU by 3 h (Kenney et al., 2025).

Such genetic down-regulation or mutations, evolved as a phage escape mechanism also profoundly reshape interactions with the host immune system (Figure 1D). The complement system can synergize with phages to create a complementary “trap” mechanism. While phages preferentially clear encapsulated sub-populations, bacteria that evade phage predation by down-regulating capsule biosynthesis genes (e.g., *pgi*) transition to a non-encapsulated phenotype. This phenotype exposes surface epitopes that facilitate enhanced complement activation, leading to increased deposition of the membrane attack complex (C5b-9), elevated membrane permeability, and consequent susceptibility to complement-mediated killing (Chen et al., 2025). This “immune exposure” underscores the therapeutic potential of combining phage therapy with innate immune effector mechanisms (Fortaleza et al., 2026).

Beyond genetic trade-offs, a direct reciprocal enhancement between phages and antibiotics underpins PAS. For instance, the synergistic effect between phage and cell-wall targeting antibiotics, like cefotaxime and meropenem, against CRAB is attributed to a dual mechanism: the antibiotic enhances phage adsorption and replication, while phage infection partially restores bacterial susceptibility to the same antibiotics (Luo et al., 2024, 2023). Recent evidence highlights a nuanced, environment-dependent synergy: in low-serum conditions mimicking the lung, the phage-derived depolymerase Dpo71 enhances ceftazidime efficacy against MDR *A. baumannii* by inducing membrane permeabilization and inhibiting oxidative phosphorylation, thereby weakening efflux and promoting antibiotic penetration (Wang H. et al., 2024). The interplay can be further modulated by the bacterial intrinsic genetic landscape. For instance, CRISPR-Cas systems in *A. baumannii* may regulate endogenous gene expression, potentially influencing the overall antibiotic resistance phenotype and adding complexity to phage-antibiotic-host dynamics (Guo et al., 2022).

*A. baumannii* also employs multiple intracellular defense systems to directly counteract phage infection. Restriction-modification (R-M) systems are among the most prevalent defense mechanisms in bacteria, cleaving incoming unmethylated phage DNA while protecting host DNA through methylation (Vasu and Nagaraja, 2013; Millman et al., 2022). Abortive infection (Abi) systems constitute another major category of defense, wherein infected cells undergo programmed cell death or growth arrest to prevent phage propagation at the cost of individual cell viability. Different mechanisms of Abi systems have been characterized, including those that form membrane pores via gasdermin-like proteins (Wein et al., 2025), deplete cellular NAD^+^ through TIR-domain NADases (Loyo and Grossman, 2025), cleave host tRNA via HNH nucleases (Ishikawa et al., 2025), or induce membrane depolarization through ion channel activation (Tan et al., 2025; Wang et al., 2025c). Toxin-antitoxin (TA) systems, including RnlAB, ToxIN, and DarTG, have also been demonstrated to function as Abi systems that are activated upon phage infection (LeRoux and Laub, 2022). Furthermore, CRISPR-Cas systems function as adaptive immune systems by integrating phage-derived spacer sequences and cleaving matching invading phage DNA (Millman et al., 2022). The presence of these anti-phage defense mechanisms in *A. baumannii* explains the rapid emergence of phage-resistant variants observed clinically and underscores the need for engineered strategies to overcome these barriers.

## 5 From mechanism to application: pharmacokinetics and optimized delivery of phage therapy

The transition of phage therapy from a concept to a viable clinical intervention requires a thorough understanding of its pharmacokinetic (PK) and pharmacodynamic (PD) properties. Unlike conventional antibiotics, phages are self-replicating entities whose *in vivo* efficacy is governed by complex interactions involving distribution, bacterial host, and immune clearance, necessitating specialized PK/PD considerations (Siopi et al., 2024).

### 5.1 *In vivo* pharmacokinetics and immune dynamics

Systemic distribution and clearance kinetics are fundamental to phage PK. Following administration in murine models, *A. baumannii* phage vB_AbaSt_W16 disseminates rapidly, reaching peak concentrations within 8 h. However, most tissues cleared the phage within 72 h, with longer retention observed in organs like the spleen (Choi et al., 2025a). A primary determinant of this clearance is the host immune system. Neutralizing antibodies and phagocytic cells of the reticuloendothelial system can rapidly sequester and inactivate phages, significantly limiting their bioavailability and therapeutic duration (Choi et al., 2026; Siopi et al., 2024). This immune-mediated clearance presents a major challenge for repeated dosing or sustained therapy. Recent strategies aim to modulate this interaction. For instance, minimal PEGylation of phage dramatically improved its PK profile, increasing plasma half-life by 2.7–3.7-fold and reducing systemic clearance by over 200-fold (Choi et al., 2026). Notably, phages exhibit non-linear pharmacokinetic behavior due to saturable uptake by the reticuloendothelial system and *in vivo* replication, meaning that traditional PK parameters such as half-life and clearance are not constant but vary with dose and infection status. This modification also attenuated pro-inflammatory cytokine responses and lowered anti-phage IgG titers, indicating enhanced immune evasion and compatibility (Choi et al., 2025a, 2026).

It should be noted that the quantitative PK data presented above are derived primarily from studies using a single phage (vB_AbaSt_W16) in a murine systemic CRAB infection model. PK parameters are expected to vary across different phage types, as evidenced by the observation that PEGylation significantly improved the PK profile of vB_AbaSt_W16 but not that of a structurally related vB_AbaSi_W9 (Choi et al., 2026). Infection site also plays a critical role. Tissue-specific PK differences have been demonstrated, with substantially longer phage retention in the spleen compared with blood or kidneys. Furthermore, infection status markedly alters PK parameters—*A. baumannii* infected mice exhibit delayed peak concentrations and prolonged half-life relative to non-infected mice (Choi et al., 2025a). Therefore, extrapolation of these findings to other phages, infection sites (particularly pulmonary or biofilm-associated infections), or clinical populations should be made with caution.

### 5.2 Pharmacodynamics, administration routes, and delivery systems

The PD of phage therapy is characterized by dose-response relationships and killing kinetics. Their ability to self-replicate at the infection site leads to exponential increases in local concentration and a dynamic, self-amplifying bactericidal effect. In a CRAB-infected murine model, phage vB_AbaSt_W16 showed a delayed time to peak concentration and elevated titers, confirming active phage replication. Such self-amplification translates into potent *in vivo* bacterial clearance, leading to a reduction of CRAB loads by 4–7 log_10_CFU/mL within 24 h in murine models (Choi et al., 2025a).

The route of administration critically influences these outcomes. For systemic CRAB infection, intraperitoneal (IP) delivery yielded higher bioavailability and more effective bacterial clearance compared to oral (PO) administration. Tools like real-time fluorescence imaging facilitate the non-invasive monitoring of *in vivo* phage distribution and dynamics for improved PK assessment (Choi et al., 2025a).

Effective delivery is crucial for therapeutic success. For localized infections such as wounds, hydrogel-based formulations have been developed to enable sustained release and improve local stability, as demonstrated by several studies. Thermosensitive hydrogels co-loaded with a phage and colistin provided sustained release, effectively killing planktonic and biofilm *A. baumannii* and showing significant *ex vivo* antibacterial activity (Mukhopadhyay et al., 2023). Similarly, carboxymethylcellulose (CMC) hydrogels containing two novel lytic phages significantly accelerated wound healing and improved survival in a CRAB-infected burn model (Elshamy et al., 2025), a result corroborated by another study using a Carbopol hydrogel loaded with a thermostable Obolenskvirus phage (Sherif et al., 2025). Beyond wound applications, hydrogel formulations have also been explored for sustained phage delivery in chronic infections, achieving up to 72-h release profiles (Wang et al., 2025b). These advanced formulations address the stability and localized delivery challenges, paving the way for topical clinical applications.

### 5.3 Clinical application strategies

Based on the interaction mechanism between phage and bacteria, current clinical application strategies to enhance the efficacy of antibacterial treatment include phage cocktail therapy, phage-antibiotic combinations, and synergistic use of endolysins with antibiotics.

A primary obstacle to the widespread adoption of phage therapy is the rapid emergence of phage-resistant bacterial mutants, which can severely compromise the efficacy of mono therapy (Siopi et al., 2024; Yuan et al., 2019). This risk is vividly illustrated by the distinct outcomes of different phages. For instance, mono therapy with phage vB_AbaP_D2 (D2) readily selects for high-frequency phage resistance in *A. baumannii*, whereas the use of phage vB_AbaS_D0 (D0) alone, or a cocktail combining D0 and D2, results in a significantly lower bacterial phage resistance mutation frequency both *in vitro* and in a murine bacteremia model (Yuan et al., 2019).

The potential of PAS is highly dependent on specific pairings. The combination of temperate phages with antibiotics such as ampicillin/sulbactam, meropenem, and colistin significantly enhances the inhibition and degradation of XDR *A. baumannii* biofilms (Rastegar et al., 2024). Specific antibiotic classes demonstrate strong synergism with phages. For instance, the phage-rifampin combination achieved a 100% survival rate in a mouse infection model, vastly outperforming monotherapy (Choi et al., 2024). Similarly, tigecycline was identified as a synergistic partner for phage vB_AbaSt_W16, with a fractional inhibitory concentration (FIC) index of 0.225, indicating strong synergy (Choi et al., 2025b). Combining phages with antibiotics such as colistin can effectively eradicate persister cells of MDR *A. baumannii*, as colistin disrupts membrane integrity and dissipates the proton motive force, thereby rendering persister cells susceptible to phage-mediated lysis (Kashyap et al., 2021; Xie et al., 2022). However, some combinations can be antagonistic. For example, while phage Indie exhibited significant synergy with ceftazidime (∑FIC=0.051), achieving over 85% bacterial reduction and overcoming phage resistance, the same phage produced an antagonistic effect when paired with piperacillin-tazobactam (∑FIC = 1.281) (Orozco-Ochoa et al., 2025). Beyond drug selection, therapeutic timing also influences outcomes. A sequential regimen, administering colistin 6 h post-phage treatment, has been shown to suppress phage resistance emergence and yield 100% survival in murine models, highlighting an optimized application strategy (Choi et al., 2025b). These synergistic approaches, spanning therapeutic and preventive applications, highlight the broad potential of integrating phage biology with other antimicrobial modalities to develop more robust and versatile strategies against *A. baumannii* infections.

Beyond specific drug pairings and timing, a broader strategic question is whether phage therapy can eventually replace conventional antibiotics. Given the current evidence, phage therapy is unlikely to completely replace antibiotics against *A. baumannii* due to the limited availability of phage banks and the inherent difficulty in rapidly screening for highly specific lytic phages against diverse clinical isolates. Most successful clinical cases have similarly used phages as adjuncts to antibiotics (Wu et al., 2021; Tan et al., 2021). Thus, a synergistic model in which phages complement existing antibiotics is currently favored.

## 6 Novel strategies and future perspectives

### 6.1 Engineered enzyme therapeutics: chimeric lysins and biofilm-targeting conjugates

**Figure 2:**
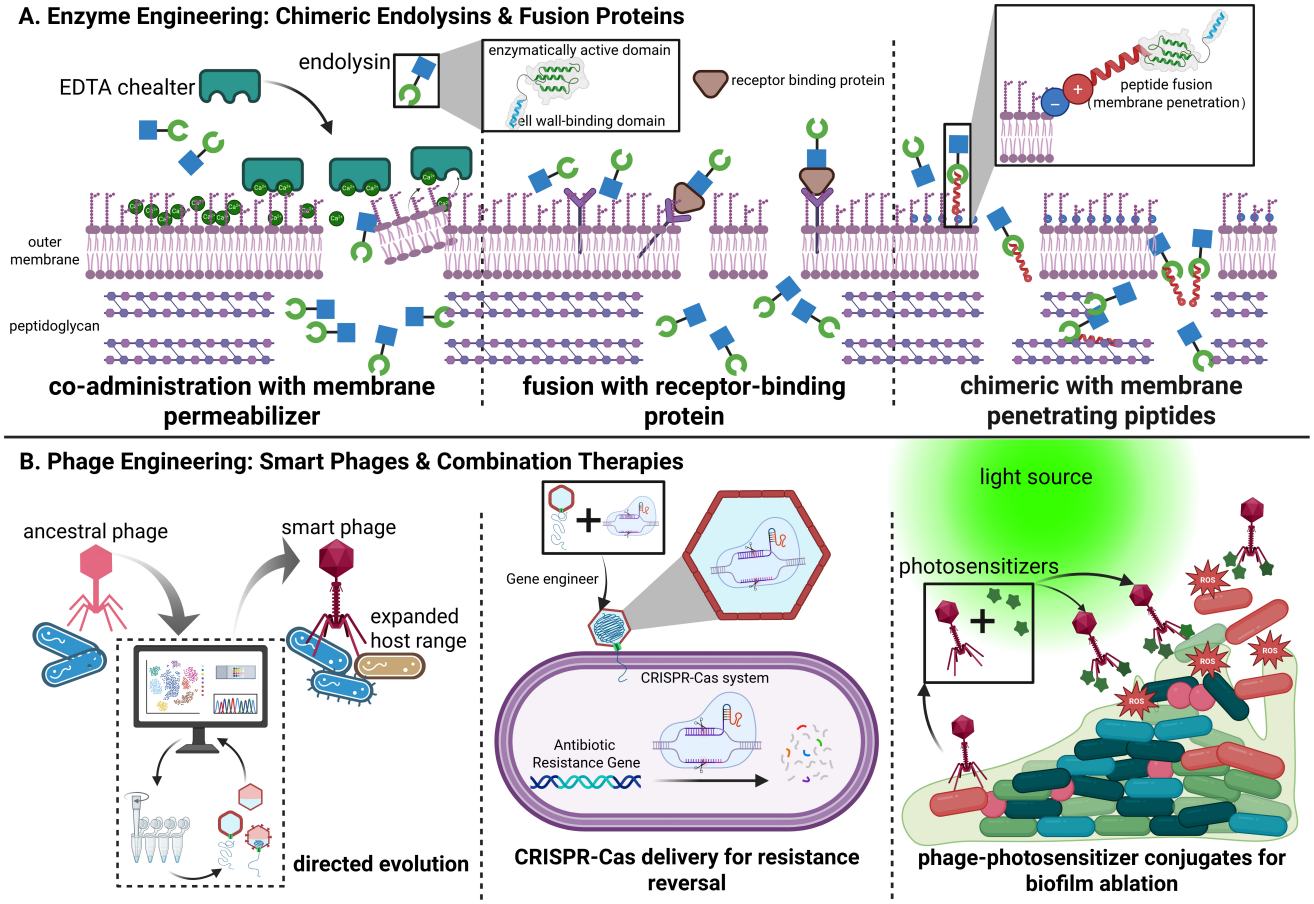
Phage and enzyme engineering strategy for therapeutics. **(A)** Enzyme Engineering: Chimeric Endolysins and Fusion Proteins. **(B)** Phage Engineering: Smart Phages and Combination Therapies. Figure was created with BioRender.com.

Phage-derived endolysins represent a promising class of enzyme-based antimicrobials due to their high specificity and low resistance induction. However, their therapeutic application against Gram-negative pathogens like *A. baumannii* is intrinsically limited by the formidable outer membrane (OM) barrier. A common strategy to overcome this barrier involves co-administration with outer membrane permeabilizers. Chelating agents such as Ethylenediaminetetraacetic acid (EDTA) or citric acid disrupt the OM by sequestering divalent cations (e.g., Mg^2+^, Ca^2+^) that stabilize lipopolysaccharide (LPS), thereby allowing the endolysin to reach its substrate (Li Y. et al., 2025; Ullah et al., 2025) (Figure 2A). Similarly, the engineered peptide-modified lysin PlyA demonstrates good activity against logarithmic-phase *A. baumannii* and *P. aeruginosa*, but its efficacy against stationary-phase cells is significantly enhanced by the addition of EDTA or citric acid (Yang et al., 2015).

To engineer next-generation lysins capable of intrinsic OM penetration, various protein engineering strategies have been employed to create chimeric enzymes, or artilysins. A primary approach involves fusing lysins with membrane-penetrating peptides (MPPs). For instance, fusing the cationic peptide CeA to the C-terminus of the globular lysin LysAB2 created LysAB2-KWK, which increased lytic activity against MDR *A. baumannii* by up to 100,000-fold and demonstrated efficacy against stationary-phase cells and biofilms (Chen et al., 2021). Similarly, fusion of the cecropin A-derived peptide to lysin OBPgp279 generated PlyA, which exhibits intrinsic activity against logarithmic-phase Gram-negative pathogens (Yang et al., 2015). Alternative fusion strategies target the OM more precisely. Fusion with phage-derived receptor-binding proteins (RBPs) or specific OM-targeting domains can guide the enzyme to the bacterial surface and facilitate translocation (Wang Y. et al., 2024) (Figure 2A). These fusions combine a catalytic domain with peptides or protein domains that disrupt or traverse the OM. The engineered phage lysin PlyKp104 achieved >5-log killing against *A. baumannii* and other Gram-negative pathogens without additional agents (Euler et al., 2023). Engineering can also exploit unique natural domains such as the S-layer homology domain in LysBT1, which enhances lytic efficacy by increasing local enzyme concentration on the cell surface (Li Y. et al., 2025).

Innovative engineering extends to combining lysins with sophisticated delivery systems. A notable example is the fusion of an endolysin with the translocation domain of botulinum neurotoxin alongside RBPs, creating a chimeric enzyme capable of broad permeation and binding, leading to significant bactericidal reduction (Park et al., 2024) (Figure 2A).

The future development of next-generation lysin therapies will focus on the rational optimization of domain combinations, the discovery of novel membrane-penetrating elements, and the integration of AI-assisted design. The goal is to create enzyme therapeutics with broader spectra, higher potency, and complete independence from auxiliary permeabilizing agents.

### 6.2 Enhancing host range and overcoming phage resistance through phage engineering and directed evolution

To overcome the inherent limitations of natural phage therapy, such as narrow host range, rapid phage resistance development, and delivery challenges, cutting-edge research is focusing on engineering phages and harnessing their components. These novel strategies aim to create more robust, predictable, and versatile anti-*A. baumannii* therapeutics.

Natural phage evolution provides a blueprint for host range expansion. An adaptive laboratory evolution study successfully generated a phage (Ab105-2phiAC1404ad) with a nearly 3-fold broader host range than its ancestor (Figure 2B). This adaptation involved rearrangements in the tail morphogenesis module, altering host receptor binding sites and even conferring a new depolymerase-expressing phenotype to the adapted phage (Blasco et al., 2022). This demonstrates the potential of directed evolution and targeted genetic modification of RBPs to tailor phage specificity against prevalent clinical strains.

A genomic survey found that *A. baumannii* strains carrying certain *cas* genes showed increased phage resistance (Leungtongkam et al., 2020). This ongoing genomic arms race, typified by the widespread co-occurrence of CRISPR-Cas systems and anti-CRISPR proteins within the *A. calcoaceticus-baumannii* complex (Mancilla-Rojano et al., 2024), provides a strategic rationale for synthetic biology. Specifically, engineering phages to deliver CRISPR-Cas systems that directly target and cleave bacterial antibiotic resistance genes or virulence factors could precisely reverse resistance and enhance bactericidal efficacy (Figure 2B).

Beyond natural phage activity, engineered strategies are being developed to enhance biofilm eradication. A notable approach combines phages with photosensitizers. For example, conjugating the phage ABP with chlorin e6 (Ce6) creates ABP-Ce6, a construct that maintains the phage's lytic function while enabling proximity-based, light-activated reactive oxygen species (ROS) production (Yu et al., 2025). This combination therapy proved highly effective against both planktonic cells and pre-formed biofilms of CRAB. Further extending the strategy of functionalizing phages with photosensitizers, Ran et al. created a photo-sensitizable phage conjugate (APNB) by covalently linking a sulfur-modified Nile Blue photosensitizer to an *A. baumannii*-targeting phage. This system exploits phage specificity for bacterial targeting and imaging, while the engineered photosensitizer enables high-efficiency ROS production. APNB effectively eradicated pre-formed *A. baumannii* biofilms *in vitro* (89.3% ablation rate) and inhibited new biofilm formation (Figure 2B). In a murine skin infection model, APNB with light irradiation promoted faster wound healing than ampicillin or polymyxin B, demonstrating its potential as a targeted combinatorial therapy against biofilm-associated infections (Ran et al., 2020).

### 6.3 Artificial intelligence in discovery, design, and personalized use of cocktail therapy

AI is revolutionizing the development of phage-based therapeutics against CRAB. By leveraging computational power, AI accelerates the discovery of novel agents and enables the design of more effective therapeutic strategies, directly addressing critical challenges such as narrow host range and rapid phage resistance emergence.

AI-driven computational mining could identify novel lytic components from vast genomic data efficiently. For example, the DeepMineLys framework (a convolutional neural network) identifies phage lysins from microbiome data. Validated on independent datasets, it achieved an F1-score of 84.00%, surpassing existing methods by 20.84%. It identified 16 lysin candidates, with 11 experimentally confirmed as active (Fu et al., 2024). Similarly, the DeepLysin software package enables the mining of putative lysins from uncharacterized phages, successfully identifying novel lysins with excellent *in vitro* antibacterial activity (Zhang et al., 2024). Such AI can screen data to discover novel depolymerases and endolysins, expanding the therapeutic arsenal against *A. baumannii* (Yang et al., 2025).

Tools like the PhageCocktail R package use algorithms (e.g., ExhaustiveSearch, ClusteringPhi) to automatically design cocktails that maximize the “expected success” (fraction of bacterial strains lysed) based on phage-bacteria interaction data (Díaz-Galián et al., 2022). This enables data-driven formulation of cocktails against diverse CRAB strains, potentially combining phages with complementary activities to enhance coverage and durability.

Furthermore, AI can accelerate phage engineering. For *A. baumannii*, machine learning could predict engineering outcomes by guiding modifications to receptor-binding proteins for broader host range, or by optimizing chimeric lysins and artilysins. AI integration is a key advancement for next-generation phage-derived enzymes and could optimize delivery systems like phage-loaded hydrogels (Liu et al., 2024).

Looking further ahead, AI might enable a paradigm shift toward personalized, on-demand therapeutics. One proposed model involves a platform integrating AI and Distributed Ledger Technology for an “instant synthetic phages” supply chain, in which AI would analyze a patient's specific CRAB isolate profile to rapidly design a tailored synthetic phage or cocktail (Pirnay, 2020). However, this model remains a conceptual proposal and currently lacks experimental validation. Nevertheless, it offers an intriguing sight into potential future directions for AI-driven personalized phage therapy.

## 7 Conclusion

The exploration of phage therapy against MDR *A. baumannii* has transitioned from a simple bactericidal concept to a multi-mechanistic and integrated strategy. A comprehensive understanding of its therapeutic mechanisms, including biofilm disruption via depolymerases, resensitization of resistant strains through receptor-mediated fitness trade-offs, the enzymatic action of endolysins and engineered chimeras, and the critical interplay with host immunity and pharmacokinetics, has laid the foundation for more rational and effective clinical application. To translate these mechanistic advances into effective clinical tools, the focus is now shifting from naturally occurring phages toward integrated, rationale-based methods that merge phages with other treatments and tailored formulations. Notable advances include better control over *in vivo* phage behavior (e.g., via PEGylation), approaches to mitigate both antibiotic and phage resistance using designed phage cocktails or combining phages with antibiotics and immune components, the development of phage-based enzymes and advanced delivery systems like hydrogels as well as the role of AI in personalized therapeutics. Achieving these clinical goals will require ongoing cooperation across fields including microbiology, structural biology, pharmacology, and clinical practice.
